# Reply to Mr. Smerdon's Strictures on the Position of Fractured Limbs

**Published:** 1821-05

**Authors:** 


*Reply to Mr. Smerdon's Strictures on the Position of Fractured Limbs.* By Dr. KINGLAKE.

THE few observations of mine which were published in the London Medical and Physical Journal for August last, have attracted the critical notice of Mr. Smerdon. The subject is certainly of sufficient importance to entitle it to all the consideration which my liberal commentator has thought proper to bestow on it. It was affirmed by me, that the question as to the preferable mode of treating fractures was still at issue; or, at least, that, in my judgment, the subject had not yet been brought to a satisfactory conclusion. It is therefore fairly open to discussion; and Mr. Smerdon has engaged in the undertaking with equal ability and candour.

Mr. Smerdon expresses much surprise at my regarding the management of fractured limbs, by placing them either in the straight or bent position, as still connected with theoretical considerations! He imagines that the luminous views taken of the subject by Pott and others, have removed all doubt on the propriety of the bent position, and have confirmed the improvement as a decided practical advantage; that theory has no concern with the obvious nature of the benefit: in fact, that it is an amendment too manifest to be reasonably denied. Much deference is due to the opinions of many of the advocates of the bent position in fractures, in the respectable list of whom Mr. Smerdon merits to be included; but yet an anxiety for the establishment of truth may be laudably indulged, in endeavouring to controvert what appears to be an unwarrantable assumption. When improvements are of such a nature as to accomplish all that could be desired, it would be equally captious and useless to resist them; but, as long as events suggest a doubt of reputed correctness, whatever may be the questioned point, it is not only allowable, but necessary, that every just objection should be freely taken against it.

Mr. Smerdon quotes the following sentence from my communication, referring to the earliest periods of practical surgery: "To have insisted at that time that laying a broken bone in a curved position, would be favourable both to the osseous union and to the eventful [eventual] straightness of the limb, would have been thought unaccountably strange, and altogether untenable." Mr. Smerdon proceeds to observe, that "Dr. Kinglake has unintentionally written this sentence very obscure." It might here be justly retorted on Mr. Smerdon, that he himself has, unintentionally, it may be also presumed, cited this sentence not very "obscure," but very obscurely. "Eventful" and eventual, in reference to the subject of inquiry, sink the meaning below obscurity, by rendering it unintelligible. Mr. Smerdon pursues his criticism by observing, that, "in several places he (Dr. K.) has very improperly made use of curved instead of bent. Now, a curve, in the common acceptation of the word, signifies a part of the circumference of a circle; but, when a joint is bent, it forms an angle more or less acute, but certainly not a curve." This objection is raised on a verbal nicety,—on an attempt at accurate discrimination,—about which it is not worth while to dispute; but it would be inexcusably wrong to admit this gratuitous assertion, by conceding the obvious mathematical truth, that any deviation from the straight line, however small, is curvilinear; and, whether it be the minutest segment of a circle or comprising a large portion of it, still it is curved or bent from the straight line. The extremities of the human body, when bent or curved at the joints, are not in a straight direction, but describe portions of a circle with angular projections.

Mr. Smerdon is too sweeping, in assuming that the bent position in fractures is almost universally adopted. The exceptions are numerous and respectable. Instances sufficiently abound, both in the metropolis and in the provinces, in public establishments and in private practice, of the straight being preferred to the bent position. It must be owned that the modern management of fractures has been powerfully influenced, and in a great measure determined, by the doctrines and recommendations of public teachers, who have felt persuaded, from anatomical as well as physiological considerations, that the bent position ought to be adopted. With this strong bias, the young practitioner has resolutely pursued the admonition given, and could have had no motive for doubting its claim to preference, until undeceived and convinced to the contrary by actual observation. With nosological references the most precise, with physiological estimations of the distinct and conjoint effects of muscular action the most profound, it has been confidently held that the straight position is fraught with constant irritation and eventual mischief; whilst bending the limb would anatomically keep at bay all obstacles, and mechanically insure the most quiescent and effectual union of the fractured bones. Notwithstanding the ample and consoling justification for the bent position, which may be derived from scientific speculations, from conditions that would appear to constitute and determine the physical necessity of the case, yet it has been often found that the straight position has been at once the refuge of the surgeon's skill and of the patient's suffering. The theoretic comforts of relaxed muscles and undisturbed bony union have been frequently tortured into the straight position, in defiance of muscular constraint, as the *solamen miseris*, as the only mode of avoiding the insufferable pains and evils of the bent posture. My own observation, and that of others, can bear testimony to the truth of this statement.

A few years since, a message was referred to me to attend a consultation with two eminent surgeons, in a case of compound fracture of the tibia: the bone had been splintered, and a point, about an inch long, of the upper end protruded through the skin; the fracture had been ably reduced, the broken ends of the bone had been placed in exact co-aptation, and skilfully bent *secundum artem*. Involuntary muscular action repeatedly displaced the broken ends of the bone, and re-produced the original protrusion. This had occurred in several instances during two days after the fracture. At the period of my attendance, which was, if my recollection be correct, on the third day after the fracture had happened, the patient was in great torture at the broken part; had been sleepless; considerable tumefaction had overspread the limb, and the protruded bone was resting on the integuments, and lapping over the other fractured end. In this state, it was proposed to take off the protruded portion of bone, with a view at once to facilitate the necessary reduction, and to obviate the excitement which its irritating point was supposed to occasion. This proposition was resisted by my suggesting that a suitable extension of the limb should be made, for the purpose of reducing the fractured bone to its natural situation, and that the bent should be changed into the straight position, with proper bandaging to keep the fractured ends in due opposition. This altered treatment, with all the ideal difficulties of muscular constraint and tension, succeeded most perfectly. No further displacement occurred of the broken bone, nor any pain that prevented refreshing sleep; the existing tumefaction speedily subsided; and, after the lapse of about six weeks, the most complete restoration of the limb was effected that could have been expected, or even desired. It may be proper to add, that the patient was a young man, of about twenty years of age, stout and muscular, and of a sanguineous temperament.

This is not a solitary instance of the beneficial effect of the practice proposed, but rather the common result of the straight position, when either originally adopted or subsequently resorted to under difficulties occurring in the bent posture.

Mr. Smerdon, with anatomical accuracy and precision, remembers and enumerates the names and actions of the muscles that are to be regulated and adjusted in the treatment of fractured limbs. He then imagines that the following sentence of mine, which he has quoted, contains a principle that is quite indefensible :—" The range of involuntary action given to the muscles connected with a fractured bone, when in a relaxed state, is such as operates most injuriously on the stationary position, in which it would be desirable constantly to confine the broken ends of the bone."—" Here," says Mr. Smerdon, " for reasons which I have before stated, I am entirely at issue with him, [Dr. K.] If distention," continues Mr. Smerdon, " be a powerful stimulus to muscular fibre, and if muscles are liable to involuntary action by being stretched, whether is it the more rational plan, in order to prevent that action, to relax them, or to place them in a state of distention ?" Here appears to lie the gravamen of the question,—Whether a relaxed or tense state of the muscular structure immediately surrounding the broken bone, be the more favourable to a steady, undisturbed position of the fractured limb ? The broken bone requires, for its speedy and uninterrupted union, the most undeviating state of mechanical rest. If this be at all disturbed, the eventual cure will be delayed, and probably rendered imperfect. Why were padding and bandaging always held to be indispensably necessary in fractures, but for the purpose of imposing on the part a constraint that should prevent any disuniting movement of the broken bone ? Mr. Smerdon conceives that " distention," or rather extension, of a muscle, is a condition of high excitement ; and that a relaxed state of that structure, is an abstraction of stimulus. This reasoning is applicable to muscular action, generally considered, but has little or no efficient relation to the muscular powers connected with the treatment of fractured bones. Nor even, generally speaking, with regard to muscular action, can it be conceded to Mr. Smerdon that extention, though a temporary, is a permanent, stimulus to the muscular fibre. It is well known that it is the property of all stimuli to have their influence diminished by frequent repetition, and much more so by constant use. This is owing to the excitability of the part acted on being so lessened as to lose its primary susceptibility for being affected. It is thus that the muscular structure connected with fractured bones soon parts with its first disposition to be inordinately excited, and at length becomes too torpidly passive to admit of being painfully stimulated by muscular extention and constraint. Muscular power exerted in a particular action for a long continuance, will be so exhausted as to incapacitate the structure furnishing it for strong contraction; so it is in the muscles that are bound up in a fracture, and laid in the straight position. Thus circumstanced, they soon become so worn and deprived of active energy, as not to occasion either pain or hurtful movement by the extended state to which they are subjected. Is it not constantly observed that the muscles of a fractured limb are, by disuse, much extenuated and enfeebled, and that they are only restored to healthy power and action by gradual and unremitted exertion? The topical constraint, therefore, that is imposed on the muscles of a fractured limb by being extended in the straight position, is highly conducive to preserving the broken ends of the bone in lineal connexion, and cannot be reasonably held to be a source of noxious irritation. The relaxed or bent position of a fractured limb certainly imparts to the contiguous muscles a sphere of motion in which involuntary and spasmodic contractions may take place, under the excitement of a broken bone, to an injurious extent; and which, in my judgment, is best obviated and provided against by the straight position.

The attempt steadily to fix the lower limb by comprehending the joints, as proposed, in the splints that may be employed, has more in it of form than reality. Unless the fixation that is applied to the joints be equally continued throughout the intermediate part of the limb, it can have no effect on the involuntary action of the muscles which are left at liberty, to be hurtfully excited by accidental occurrences. In fact, the main curative indication in the management of fractured limbs, is to avoid all motion of the broken bone, whether from adventitious causes or from muscular action, that might derange the lineal connexion of the severed ends, and induce unequal union and ultimate deformity. The constraint of mechanical confinement, carried no further than would be quite consistent with an unimpeded circulation through the bandaged part, and with the degree of pressure that would securely restrain, but in no measure paralyze, muscular power, is indispensably requisite, and most effectually insures early ease, progressive amendment, and, finally, the best attainable restoration of the fractured limb.

Although anatomical and physiological disquisitions may be plausibly indulged on the subject of the muscular structure, instead of the broken bone, and the regulation of its power be regarded as the leading object, yet practical experience has sufficiently ascertained the visionary and fallacious nature of this scientific reasoning, and has satisfactorily proved that an extended limb may be more quietly and immoveably stationed for weeks together in that position, than could be effected by the bent posture. If this be the case, and of the truth of which there does not appear to be any doubt, the question may be tenaciously insisted on and ingeniously argued, but still its real merits will remain indisputable; and it is an object of great importance that its just claims should be recognized and allowed.

My observation may have been less extensive than that of Mr. Smerdon, but it has been fully sufficient to satisfy me that both the management and result of the straight position in fractures are far more advantageous than in the bent situation. Generally speaking, the attending inconvenience is less, and the restoration of the fractured limb more natural, in the former than in the latter mode of treatment.

It may be justly hoped, in the spirit of Mr. Smerdon's observation, that the equally cruel and reprehensible practice of periodically moving the broken ends of a fractured bone, to ascertain if the desired union had taken place, is by no means general, and that surgeons of intelligent reflection would deservedly reprobate it. But still it does occur; and, were occasions for noticing it so frequent with others as with myself, no question could be raised as to the existence of the mal-practice. It has even occurred to me to see it pursued in a public hospital, by surgeons of reputed skill and eminence, and that, too, with immediate and ultimate effects deplorably injurious. Binding up compound fractures after an exact reduction of the displaced bones, with suitable strictness, and laying the limb in the straight position, would appear to afford the best prospect of a speedy, an undisturbed, and an effectual cure. Effusions, suppurations, sloughings, and exfoliations, consequent on unnecessary exposure and the usual applications to the wounded part, are best avoided by effecting, under moderate pressure from bandage, an union of the soft parts by the first intention. This event, under the comparative quietude and stillness afforded by the straight position, may be generally accomplished in a manner equally gratifying to the feelings of the patient and to the reputation of the surgeon.

It is not to be inferred, because a preference would seem to be due to the straight position of fractured limbs, that, with vigilant and judicious management, the bent position may not also succeed very happily. The provisions of nature for reuniting broken bones are so abundant and so efficiently operative, that mechanical obstacles only can impede the salutary progress of the natural work. The surgeon's province is to facilitate, by local arrangements, the process of osseous union; and adequate experience and dispassionate reflection will best enable him to make a correct election between the two positions offered to his preference. It is possible that the two postures may be a mutual resource for each other,—that changing from the one to the other, under circumstances of unappeasable irritation, might afford relief. It is not in my power to speak from observation of the benefit of the alternative from the straight to the bent position; but of that from the latter to the former, it may be truly asserted that immediate and progressive advantage will be found, if not uniformly, in most instances, to result from it. The evils of the bent position arise from the unconfined and almost incoercible motion of the broken bones and surrounding muscles. Insufficiently restricted, these parts are liable to hurtful movements. In the straight position, with a moderately strict bandage extended over the limb, somewhat concentrating its pressure on the fractured part, all disconcerting and painful movements of either bones or muscles will be effectually prevented; and, after the usual lapse of time, the limb will be restored to its natural shape and power of motion.

*Taunton; Feb.* 28, 1821.

